# Reducing maternal and newborn mortality in Nigeria—a qualitative study of stakeholders’ perceptions about the performance of community health workers and the introduction of community midwifery at primary healthcare level

**DOI:** 10.1186/s12960-019-0430-0

**Published:** 2019-12-23

**Authors:** Ekechi Okereke, Salisu Mohammed Ishaku, Godwin Unumeri, Bello Mohammed, Babatunde Ahonsi

**Affiliations:** 1 Population Council, 16 Mafemi Crescent, off Solomon Lar Way, Utako, Abuja, Nigeria; 2 World Health Organization (WHO), Abuja, Nigeria; 3 UNFPA China, 1-161 Tayuan Diplomatic Office Building, Beijing, China

**Keywords:** Community midwifery, Primary healthcare, Maternal and newborn mortality, Nigeria

## Abstract

**Background:**

Rural communities in Nigeria account for high maternal and newborn mortality rates in the country. Thus, there is a need for innovative models of service delivery, possibly with greater community engagement. Introducing and strengthening community midwifery practice within the Nigerian primary healthcare system is a clear policy option. The potential of community midwifery to increase the availability of skilled care during pregnancy, at birth and within postpartum periods in the health systems of developing countries has not been fully explored. This study was designed to assess stakeholders’ perceptions about the performance of community health workers and the feasibility of introducing and using community midwifery to address the high maternal and newborn mortality within the Nigerian healthcare system.

**Methods:**

This study was undertaken in two human resources for health (HRH) project focal states (Bauchi and Cross River States) in Nigeria, utilizing a qualitative research design. Interviews were conducted with 44 purposively selected key informants. Key informants were selected based on their knowledge and experience working with different cadres of frontline health workers at primary healthcare level. The qualitative data were audio-recorded, transcribed and then thematically analysed.

**Results:**

Some study participants felt that introducing community midwifery will increase access to maternal and newborn healthcare services, especially in rural communities. Others felt that applying community midwifery at the primary healthcare level may lead to duplication of duties among the health worker cadres, possibly creating disharmony. Some key informants suggested that there should be concerted efforts to train and retrain the existing cadres of community health workers via the effective implementation of the task shifting policy in Nigeria, in addition to possibly revising the existing training curricula, instead of introducing community midwifery.

**Conclusion:**

Applying community midwifery within the Nigerian healthcare system has the potential to increase the availability of skilled care during pregnancy, at birth and within postpartum periods, especially in rural communities. However, there needs to be broader stakeholder engagement, more awareness creation and the careful consideration of modalities for introducing and strengthening community midwifery training and practice within the Nigerian health system as well as within the health systems of other developing countries.

## Introduction

Alongside inadequate financing, essential medicines supply gaps and a myriad of service delivery challenges, the inadequate number of skilled healthcare workers is a major challenge to providing quality healthcare services as well as for the attainment of the health-related sustainable development goals (SDGs) [1, 2]. The shortage in the health workforce, especially within low- and middle-income countries, and the increasing evidence that community health workers contribute to improving health outcomes is generating new interest for more community health worker initiatives [3]. Many developing countries are making concerted efforts to increase the production and distribution of their healthcare personnel [4], including through introducing new cadres of community health workers [5]. Community health workers have different names, with many working within the community while some have facility-based tasks. Many of these community health workers work in the healthcare system voluntarily without receiving a salary, but some cadres are in paid employment [3].

At primary healthcare (PHC) level in Nigeria, community health workers who are in paid employment comprise mainly community health officers (CHO), community health extension workers (CHEWs) and junior community health workers (JCHEWs). According to the National Guidelines for the development of primary healthcare systems in Nigeria, the JCHEWs are expected to provide basic and essential community services with guidance from standing orders^1^ as well as spend about 90% of their time working within the community. The community health functions of JCHEWs include ensuring the community’s participation in health-related activities, conducting home and community visits, clients’ follow-up and identifying and registering pregnant women for antenatal care and support. The CHEWs according to the National Guidelines have similar functions as the JCHEWs but are expected to spend about 40% of their time working in the community and more time working within health facilities, promoting preventive maternal and child healthcare and managing clients according to standing orders. The community health officer has more administrative roles within the health facility, but in addition has similar community and maternal child healthcare responsibilities as the JCHEWs and CHEWs [6]. These types of frontline healthcare workers, with strong ties to the community, are less likely to be influenced by the dynamics of international health labour markets [7]—thus less likely to engage in inter-country health workforce migration.

About 99% of maternal, newborn and child deaths occur within developing countries [8, 9]. Nigeria which is the most populous country within sub-Saharan Africa, similarly has poor maternal, newborn and child mortality indices [10]. The country has a maternal mortality rate (MMR) of 576 deaths per 100,000 livebirths and estimates which indicate that maternal deaths are responsible for about a third of all deaths among women of reproductive age [11]. The situation is much worse within the northern parts of the country, where the MMR is estimated to be over 1000 deaths per 100 000 live births [12]. Nigeria’s infant mortality and under-5 mortality rates are estimated at 69 deaths per 1000 live births and 128 deaths per 1000 live births respectively [11]. The maternal, newborn and child health indices in Nigeria are typically worse within rural areas. For example, the maternal mortality rate is estimated at 828 deaths per 100 000 live births in rural areas in contrast to 351 deaths per 100 000 live births in urban areas [13].

There is evidence that community health workers contribute towards the delivery of maternal, newborn and child health (MNCH) services such as childhood immunization, antenatal care and facility-based deliveries within PHC settings [14]. Furthermore, the implementation of the task shifting and sharing policy has empowered many community health workers, through training and capacity building, to acquire skills which are crucial for promoting maternal, newborn and child health in Nigeria [15]. However, there is limited evidence about the performance of community health workers vis-a-vis maternal, newborn and child health service delivery in Nigeria. As a result, it is difficult for policy makers to ascertain what specific changes to the health workforce maybe required to significantly improve health outcomes.

The human resources for health (HRH) project, implemented by Population Council and the World Health Organization in Nigeria, carried out a study to assess different stakeholders’ perceptions about the performance of community health workers who are in paid employment in Nigeria, with Bauchi and Cross River States as study sites. The study also assessed whether there is a case for introducing community midwifery as a new mid-level cadre in paid employment within PHC settings in Nigeria. Following the commencement of community midwifery practice in some states within northern Nigeria, this study was designed as part of the consultative process prior to the introduction of a community midwifery programme within Bauchi State College of Nursing and Midwifery and the commencement of community midwifery practice within Bauchi as well as possibly within Cross River State.

## Methods

### Study sites

This study was carried out in Bauchi State in the North-eastern part of Nigeria which has a predominantly Muslim population as well as in Cross River State which has a predominantly Christian population within the Southern part of the country.

### Study design, study population, and sampling strategy

Key informant interviews within a qualitative study design were used to elicit the required information from the study participants using appropriate key informant interview (KII) guides. These KII guides were first developed, pilot-tested and finalized for use by the study researchers. The study population consisted of 44 policy makers and healthcare managers, i.e. stakeholders selected from a range of institutions in Bauchi and Cross River States. The study population consisted of senior officials within Ministries of Health and other health-related parastatals such as the State Primary Healthcare Development Agencies, Heads of health training institutions, key officials of Local Government Health Departments, Managers from health development organizations and officials from associations of frontline health workers within both Bauchi and Cross River States. (Please see Table 1 for breakdown of policy makers and healthcare managers interviewed for the study). A purposive sampling approach was employed to select key informants for the study, based on their knowledge and/or experience working at the PHC level. The purposive sampling technique was appropriately selected as the sampling strategy to ensure that all selected key informants from both study states had a minimum of 5 years work experience with the Nigerian PHC system, covering areas such as training and capacity building of health workers, service delivery and PHC governance, thus making them well-suited to offer rich insights for addressing the study objectives.

**Table 1:** Breakdown of policy makers and healthcare managers interviewed for the study within the two states in Nigeria

S/N	Category of stakeholders interviewed for the study	Number of stakeholders		
		Bauchi	Cross River	Total
1	Head of Health Training Institution	3	7	10
2	Senior Ministry of Health Official	4	2	6
3	Senior Official from Health-related parastatal within the State e.g. from the State Primary Healthcare Development Agency, State Hospital Management Board etc.	8	5	13
4	Local Government Health Department official (e.g. Primary Healthcare (PHC) Director, Maternal and Child Health Coordinators) within Local Government Areas in study states	4	4	8
5	Manager in Health Development Organization	2	3	5
6	Official from frontline health worker association	1	1	2
Total		22	22	44

### Data collection and data management

Interviewers with expertise in conducting key informant interviews as well as understanding of the context and culture of the study states were recruited. For quality assurance purposes, the interviewers were trained by qualitative research methodology experts on the use of the key informant guides. In addition to role plays and participating in practical exercises, they were retrained on research ethics*.* Prior to commencing data collection, written informed consent was sought and obtained from each study participant.

Data collection using key informant interview guides took place starting in November 2015 and continued through the first and second quarters of 2016. To ensure validity, the KII guide was reviewed and quality-assured by senior health experts from the Bauchi and Cross River States Ministries of Health and researchers from the University of Ibadan in Nigeria, who provided support during the design of the study. The KII guide consisted of broad questions and follow-up probes on the performance of community health workers in maternal, newborn and child healthcare service delivery and specific actions that could be taken to improve or enhance MNCH service delivery effectiveness.

The interviews were recorded using a tape recorder. Consent to be tape-recorded was sought and obtained from each respondent as part of the informed consent process. After the interviews, the interviewers transcribed the audio recordings verbatim, and the transcripts and audio recordings were sent to Population Council Country office in Nigeria for archiving. The transcripts were subsequently compared with the audio recordings to ensure completeness. A pre-determined coding framework was applied, and subsequently thematic analyses of the data were undertaken. Members of the study team who were involved during the data collection process also provided assistance in categorizing the data. Regular meetings were held by the study team in order to reach consensus on the interpretation of the data collected during the interviews. The study focused on the investigation of the perspectives of PHC managers and policy makers (rather than clients) as key stakeholders since their inputs would largely shape any official efforts at reforming or improving the performance of the Nigerian PHC system.

### Ethical considerations

Ethical approval was granted by Bauchi and Cross River States’ Research Ethical Committees and Population Council’s Institutional Review Board (IRB). The study was conducted based on set ethical guidelines, and informed consent was obtained from each study respondent before each interview commenced. Study respondents were assured of the confidentiality of their responses. Only generic descriptions and the location of each key informant were recorded by the researchers without explicitly identifying particular key informants vis-à-vis their specific responses or statements.

## Results

### Performance of community health workers in maternal, newborn and child health service delivery

Stakeholders’ perceptions of community health workers’ performance were varied—some felt that the community health workers were having a positive impact on maternal and child morbidity and mortality while others indicated that their overall performance is suboptimal. Study respondents however indicated that these community health workers provide services amidst difficult circumstances within rural settings including lack of basic amenities, inadequate infrastructure and poor working conditions.

“I would say their performance has reduced morbidity and mortality among the newborn and under-fives because of immunization…you now see that you don’t have many of these childhood diseases”

**Manager from non-government organization [NGO], Cross River**

“Though they are trying but the overall performance is still low…when you compare their performance with [health] outcomes, they are not doing enough in terms of meeting the needs of maternal and child health”

**Manager from non-government organization [NGO], Bauch**i

Many respondents, especially in Bauchi state which has a predominantly Moslem population indicated that with respect to maternal and child healthcare, women tend to perform better. Some suggested that because of religious and cultural norms that restrict male-female interactions particularly in northern Nigeria, women tend to be more effective community health workers than men.

“…services of women in maternal and child health are better than that of men.”

**Senior official in Health-related parastatal, Bauchi**

“…most of the clients in rural areas are women and they [female community health workers] find it easy to mingle with them and even visit them at their homes.”

**Senior official, Ministry of Health, Bauchi**

Among the roles that were identified as worst performed by community health workers include record keeping and research. It was mentioned that community mobilization was not adequately performed. In addition, post-natal care services are not being performed as expected.

“The role they perform worst is that they don't do research…or maybe even if they do, the government doesn't implement…”

**Head of Health Training Institution, Bauchi**

“Well I think they have problems in the post-natal services…for any woman or pregnant woman, they should have a register. They know her location, they know her address and after deliveries, maybe they should be looking at their register and tracking, because you are expected to come back to the health facility and if she doesn’t come, they should have a way of contacting her”

**Senior official, Ministry of Health, Bauchi**

“Another role that they are expected to play is community mobilization, which currently is poorly performed…”

**Senior official in Health-related parastatal, Cross River**

Some key informants mentioned that some of the community health workers attend to cases which they are not adequately trained or skilled enough to manage, in many instances without adequate supervision. These cases when not treated well or referred to higher levels of care on time, lead to increases in the maternal, newborn and child mortality, especially in rural communities.

“…the JCHEWs and CHEWs are handling cases they are not supposed to handle, because the skills are not just there!”

**Senior official in Health-related parastatal, Bauchi**

Some study participants were of the view that community health workers sometimes delay the referral of cases. Some stakeholders asserted that if community health workers make timely referrals, many of the cases with complications referred to higher levels of healthcare from PHC level will be avoided.

“…if they notice something they cannot do and refer, those problems wouldn’t be there, delayed referral is a big problem”

**Official from association of frontline health workers, Cross River**

### Introducing community midwifery to provide maternal and newborn services in PHC settings

Participants expressed mixed views concerning the need to introduce community midwifery to deliver maternal and newborn health services in PHC settings within Nigeria. Some respondents felt that creating a new cadre will potentially increase the number of health workers available to provide healthcare services to patients and clients.

“If at all there is opportunity to do so, yes and this will make us to have additional staff…so if at all they create another cadre for these things it will be better”

**Local Government Health Department official, Bauchi**

“There is need for community midwifery, because there is a gap in manpower, so if we have community midwifery at least it would then help in bridging the gap…”

**Senior official, Ministry of Health, Bauchi**

Another potential benefit of introducing community midwifery according to some respondents is to further reduce maternal and child mortality rates as these cadres will be recruited to work in rural communities, where the morbidity and mortality rates are typically high. Some key informants felt that introducing community midwifery will potentially increase skilled birth attendance during delivery and postpartum periods, particularly within rural communities. It was also suggested that trained community midwives should return to their communities to reside in and provide maternal and newborn health services upon completion of their studies.

“The benefits are that they will be posted to their rural areas and they would be posted to their local government area, they will be there, they will stay with the people, if there is any problem, they will be called upon to come and assist…”

**Senior official, Ministry of Health, Bauchi**

Some respondents (especially from Bauchi) were very particular about the gender of those to be trained as community midwives. It was mentioned that this cadre should be mostly (possibly only) females based on the existing cultural and religious norms prevalent within northern Nigeria

“…if you should have a new cadre, they should be mostly women because looking at the culture of the people [within northern Nigeria], it is only a female that can enter everybody’s house, a male has limits to his relative’s house…”

**Manager from non-government organization [NGO], Bauchi**

However, others felt that introducing community midwifery at the PHC level may lead to duplication of duties among the health worker cadres, possibly creating disharmony.

“…there may be rivalry between the cadres and you know once you have this, you could have acrimony between staff and that will not be good for healthcare services”

**Senior official in Health-related parastatal, Bauchi**

“…it will end up creating enmity and disharmony among the health personnel”

**Official from association of frontline health workers, Bauchi**

Some study respondents believed that instead there should be concerted efforts towards training and retraining the existing cadres of health workers to provide the required maternal and newborn services.

“…there is no need [to introduce community midwifery], let us utilize the ones we have…”

**Official from association of frontline health workers, Bauchi**

“…there is really no need to create new cadres for health workers but rather we should look at improving the skills and efficiency of the ones we already have. If there are new innovations in the areas of maternal and newborn service delivery, we should try to introduce those new skills to the healthcare workers we have and then equip them”.

**Head of Health Training Institution, Cross River**

Some study respondents indicated that currently there are a lot of the existing cadres of community health workers who have been trained but remain unemployed. They argue that these unemployed health workers should be employed to bridge the gaps in human resources for health, instead of introducing community midwifery as a new cadre at the PHC level.

“…In Bauchi state, we have one public school of health technology which has produced a lot of CHEWs and JCHEWs who are yet to be employed”

**Senior official, Ministry of Health, Bauchi**

“…I don’t think it is necessary, we need to develop and absorb the existing ones…because the ones that are already in the field we are not making use of them”.

**Head of Health Training Institution, Cross River**

Other study respondents suggested that rather, the government should build the capacity of the existing community health workers by effectively implementing the task shifting and sharing policy or by revising the training curriculum of these community health workers, so that these cadres can provide the required services and thus fill the gaps identified.

“To the best of my knowledge, the answer is no, because all we need is to redesign the curriculum for the training of community health workers so that they can handle those things needed to take care of mothers and children.”

**Head of Health Training institution, Cross River**

“…what I rather think is that the curriculum of the CHEWs and JCHEWs should be revised and they should include some aspects of midwifery…”

**Official from association of frontline health workers, Cross River**

## Discussion

The study assessed different stakeholders’ perceptions about the performance of community health workers who provide maternal, newborn and child health services at the PHC level as well as considered possible justifications for introducing community midwifery as a new cadre in paid employment within PHC settings, using Bauchi and Cross River States in Nigeria as study sites. This study, as part of the stakeholders’ engagement process, is important especially since the introduction of community midwifery into the health systems of other developing countries, e.g. Kenya, led to an increase in women’s access to skilled care during pregnancy, childbirth and postpartum periods [16].

Feedback from different stakeholders about the performance of community health workers was mixed—while some respondents believe that the services of the community health workers are helping to address maternal, newborn and child mortality, others feel that much more still needs to be done. Females were reported as being better performing community health workers, especially within the northern parts of the country where due to religious and cultural norms, they enjoy easier access to women and children within the community. There are also strong arguments that to improve the effectiveness of the work of community health workers, it is important and necessary to better integrate the private health sector within the overall PHC system in Nigeria [17].

There were contrasting views about the need to introduce community midwifery within the PHC level in Nigeria. Study respondents from Bauchi State were more inclined to support community midwifery within their healthcare system while most respondents from Cross River expressed their reservations about introducing community midwifery. For those supportive of introducing community midwifery, they argue that this will help bridge the existing human resources for health gaps especially at the PHC level. They suggest that introducing community midwifery is required to address the high maternal and newborn mortality within the northern parts of the country and especially so within rural communities in northern Nigeria [18, 19]. The Midwives Services Scheme (MSS), a maternal and newborn healthcare intervention targeted at primary healthcare level, was similarly introduced by the Federal Government of Nigeria to address high maternal and newborn mortality, especially within rural areas in Nigeria [20]. Furthermore, the “three delays” are known barriers for women and children trying to access maternal, newborn and child health services [21–23]. The introduction of community midwives within rural communities could be a good maternal and newborn healthcare intervention to minimize the impact of the three delays and thus improve maternal and newborn health outcomes.

Some States, mainly within northern Nigeria, have commenced community midwifery education in their health training institutions, including Kano and Zamfara States. Community midwifery education ensures that a community midwife develops the skills for normal midwifery practice but with emphasis on primary healthcare. First, there is the selection of young girls and women from different rural communities with the assistance of their respective community leaders. These girls and young women typically have an observed or expressed interest in healthcare service delivery. These girls/young women are then enrolled as trainee community midwives within health training institutions such as Colleges/Schools of Nursing and Midwifery which offer community midwifery programmes. There is the clear understanding that the community midwives will return to serve within their local government areas, following the completion of their training.

Student intake is typically at a maximum of 50 students per academic session, but this can be adjusted to comply with the tutor/student ratio and other student enrollment policies and practices within the health training institution. In some of the States, about two students are selected from “participating” local government areas per session and only students that provide evidence of residence within their respective local government areas are indexed as trainee community midwives.

The community midwifery training programme is for a period of 18 months, and upon successful completion, there is 6 months’ compulsory community practice. The successful student then registers to practice as a community midwife within Nigeria *only*.

Most of the states within northern Nigeria which have commenced the community midwifery programme have recently graduated their pioneer enrollees, and these recent graduates have been deployed to their respective rural communities to provide community midwifery services. These community midwives are recognized as a cadre of community-focused skilled birth attendants, deployed to tackle the strikingly high maternal and newborn morbidity and mortality within northern Nigeria. Interestingly, most states within the southern part of Nigeria have not commenced and do not appear to be actively considering community midwifery programmes within their health training institutions. Currently, community midwifery practice is also non-existent within southern parts of Nigeria.

Most respondents who expressed reservations about introducing community midwifery suggested that such a strategy could lead to duplication of duties, rivalry among cadres and disharmony among health workers working at the PHC level. Instead, it was stated that more of the existing cadres of community health workers should be employed. Also, these community health workers should be trained and retrained to strengthen their skillsets via the effective implementation of the task shifting and sharing policy in Nigeria. Furthermore, it was proposed that there should be revisions to the training curriculum of community health workers, possibly introducing aspects of midwifery as well as other key components of maternal, newborn and child health, instead of introducing an entirely new cadre to address these MNCH challenges.

Majority of the stakeholders from Cross River State were not supportive of introducing community midwifery as a new cadre in contrast to most stakeholders from Bauchi State, perhaps a reflection of the greater sense of urgency especially for northern Nigerian States in the face of their much higher levels of maternal and child mortality. The World Health Organization (WHO) typically does not promote the use of traditional birth attendants [24]—the WHO regards traditional birth attendants as unskilled birth attendants. However, the WHO is more likely to welcome the introduction of community midwifery as a new cadre of community-focused skilled birth attendants—potentially accepting the cadre as a good substitute for traditional birth attendants within rural community settings. Since community midwives are in paid employment, there is need to undertake a proper evaluation of the economic costs of introducing community midwifery (with its potentially significant financial implications) against the benefits of improved maternal and newborn health outcomes within the Nigerian PHC system.

## Conclusion

Stakeholders’ feedback about community health workers’ performance was mixed. Stakeholders from Bauchi State expressed more willingness to introduce community midwifery than those from Cross River State. Ultimately, introducing female community midwives with strong ties to the community and having easy access to most families within the communities could be of some added value amidst efforts to address high maternal and newborn mortality within rural northern Nigeria. However, there is need for a careful assessment of the modalities and benefits of introducing community midwifery into the Nigerian healthcare system and possibly in the health systems of other developing countries to address high maternal and newborn mortality rates.

## Data Availability

**T**he datasets for this study are available from the corresponding author upon reasonable request.

## Abbreviations

CHEW: Community health extension worker
CHO: Community health officer
FLHW: Frontline health worker
GAC: Global Affairs Canada
HRH: Human resources for health
IRB: Institutional Review Board
JCHEW: Junior community health extension worker
KII: Key informant interview
LGA: Local government area
MMR: Maternal mortality rate
MNCH: Maternal newborn and child health
NGO: Non-governmental organization
PHC: Primary health care
SDGs: Sustainable Development Goals
WHO: World Health Organization

## Footnotes

1 ‘Standing orders’ is a document used by community health workers which outlines the protocol for dealing with clients and patients based on national clinical guidelines, especially at a primary healthcare level in Nigeria.
